# Myofascial Structural Integration Therapy on Gross Motor Function and Gait of Young Children with Spastic Cerebral Palsy: A Randomized Controlled Trial

**DOI:** 10.3389/fped.2015.00074

**Published:** 2015-09-10

**Authors:** Elizabeth C. Loi, Christina A. Buysse, Karen S. Price, Theresa M. Jaramillo, Elaine L. Pico, Alexis B. Hansen, Heidi M. Feldman

**Affiliations:** ^1^Department of Pediatrics, Stanford University School of Medicine, Palo Alto, CA, USA; ^2^Advanced Rolfing, Private Practice, Palo Alto, CA, USA; ^3^Physical Therapy and Rehabilitation Science, University of California San Francisco, San Francisco, CA, USA; ^4^Pediatric Rehabilitation, Santa Clara Valley Medical Center, San Jose, CA, USA; ^5^Department of Family Medicine, Providence Milwaukie Hospital, Milwaukie, OR, USA

**Keywords:** cerebral palsy, spasticity, children, myofascial structural integration, Rolfing^®^, motor function, gait

## Abstract

Though the cause of motor abnormalities in cerebral palsy is injury to the brain, structural changes in muscle and fascia may add to stiffness and reduced function. This study examined whether myofascial structural integration therapy, a complementary treatment that manipulates muscle and fascia, would improve gross motor function and gait in children <4 years with cerebral palsy. Participants (*N* = 29) were enrolled in a randomized controlled trial (NCT01815814, https://goo.gl/TGxvwd) or Open Label Extension. The main outcome was the Gross Motor Function Measure-66 assessed at 3-month intervals. Gait (*n* = 8) was assessed using the GAITRite^®^ electronic walkway. Parents completed a survey at study conclusion. Comparing Treatment (*n* = 15) and Waitlist-Control groups (*n* = 9), we found a significant main effect of time but no effect of group or time × group interaction. The pooled sample (*n* = 27) showed a main effect of time, but no significantly greater change after treatment than between other assessments. Foot length on the affected side increased significantly after treatment, likely indicating improvement in the children’s ability to approach a heel strike. Parent surveys indicated satisfaction and improvements in the children’s quality of movement. MSI did not increase the rate of motor skill development, but was associated with improvement in gait quality.

## Introduction

Cerebral palsy (CP) is the term used to describe a set of non-progressive disorders of movement and posture that lead to limitations in functional activity and can be attributed to disturbances that occurred in the fetal or infant brain. These disorders of movement are frequently accompanied by other medical or functional issues: disturbances of sensation, perception, cognition, communication, and/or behavior; epilepsy; and/or secondary musculoskeletal problems (1). The prevalence of CP has been reported to be 2.11 (2) to 3.6 cases/1000 (3), making it the most common movement disorder of childhood. The most prevalent variant is spastic CP. Muscles affected by spasticity have increased velocity-dependent sensitivity to stretch, causing stiffness, tightness, and interference with movement, which can lead to joint contracture and deformity. Children with mild forms of spastic CP usually walk independently. Visual gait analysis has identified slower velocity, shorter stride length, and longer double support time in ambulatory children with CP relative to typically developing peers (4).

None of the common treatments for spastic CP, including physical and occupational therapy; bracing; medications; and surgical procedures is completely satisfactory. Non-invasive treatments often have limited or temporary treatment effects that fail to substantially change the natural course of the condition or improve an affected limb’s functional capacity (5, 6). Localized treatment with injections of botulinum toxin A is associated with only temporary improvements in selected gait parameters and in reducing spasticity (7–10). Systematic reviews suggest that a combination of approaches may be more effective than a single approach to treatment (11).

In the past, spasticity was attributed solely to the etiologic non-progressive central nervous system injury. Recent evidence suggests that skeletal muscle and its extracellular matrix are altered in CP (12, 13). Increased passive stiffness within the muscle bundle is accompanied by increased collagen content within the extracellular matrix and by increased sarcomere length, rather than by abnormalities of the mechanical properties of individual muscle fibers. These findings implicate structural changes in the muscle and extracellular matrix of muscle as important contributors to the increased muscle tone and stiffness in CP (14). Targeting the local structural changes, especially before the development of contractures and deformities, might, therefore, be a helpful component of a comprehensive strategy to improve motor function in young children with CP. The goal of the present study was to determine if a treatment that manipulated muscle and surrounding fascia, used as an adjunct to physical therapy, occupational therapy, and routine medical care, could improve motor function and gait in young children with spastic CP.

The treatment we assessed was myofascial structural integration (MSI). MSI is a specific manipulative and movement-based complementary medicine practice, developed by Dr. Ida P. Rolf in the 1930s. In MSI, a trained practitioner manually manipulates muscle and fascia in order to free up stiff body parts and joints, increase stability, improve balance and mobility, and put the body into proper alignment to facilitate improved motor patterns. This treatment was selected because its goals are appropriate for children with CP, it follows a standard and reproducible 10-week protocol and it addresses the whole body, not just legs or arms. Previous research on MSI for individuals with CP found that patients with mild CP experienced improvements in such gait parameters as cadence, velocity, and stride length after a standard course of treatment with MSI (15). A recent preliminary randomized crossover design trial of MSI in young children with CP showed greater improvements after a course of MSI than after a comparable number of control play sessions, though some children improved throughout the entire study (16). A case report found improvements in cadence and double support time in two children with spastic CP after MSI (17). MSI is distinct from massage, another movement-based complementary practice, which has been shown to have variable effects on adolescents with CP (18).

The primary aim of this study was to determine whether MSI therapy, used as an adjunct to physical and occupational therapy, could improve gross motor function in young children with spastic CP. We hypothesized that a course of MSI treatment added to the existing regimen of physical and occupational therapy would lead to greater gains in gross motor function than the therapies alone. A secondary aim of the study was to evaluate changes to select gait parameters after the treatment in those children who were ambulatory at the time of Enrollment. The final aim was to obtain parental impressions of the MSI treatment and its impact on the children after treatment. Though MSI is a non-invasive approach, we surveyed families after the treatment to determine if there were any adverse effects and to learn if parents saw benefits that were not captured in our assessments.

If MSI were to prove effective, it could be incorporated into the treatment plan of young children with spastic CP as an adjunct to conventional therapies. Ideally, it would delay or eliminate the need for risky, invasive, or time-limited treatment options. Because it is safe, it could also be used repeatedly across the child’s development. It is important to prove efficacy because though safe, the standard course of 10 sessions may not be reimbursed by health insurance and is, therefore, expensive for families.

## Materials and Methods

### Participants

Young children with spastic CP were recruited from a university medical clinic, state-funded medical therapy programs that serve children with CP, and through self-referral from local publicity. We studied young children, less than age 48 months at the time that consent was obtained because they are in a dynamic period of development, generally before the onset of deformities and contractures, and therefore possibly more likely to change than older children or adults. Eligibility criteria were as follows: diagnosis of spastic CP (or mixed CP with spasticity); no recent history of seizures, surgery, or botulinum toxin A injections; receiving physical therapy; and level II, III, or IV on the Gross Motor Function Classification System (GMFCS) or a level of II, III, IV on the Manual Ability Classification System (MACS) for children who were GMFCS I, as determined by a physical therapist or pediatrician on the study team. Throughout the study, the children continued to participate in their typical treatment regimen, which included physical therapy at minimum and depending on the child, may have also included occupational therapy, medications, other complementary treatments (e.g., Feldenkrais Method), and regular recreational activities (e.g., swimming). The Institutional Review Board at Stanford University approved the protocol and parents provided informed consent prior to their child’s participation.

### Study design

**Figure 1:**
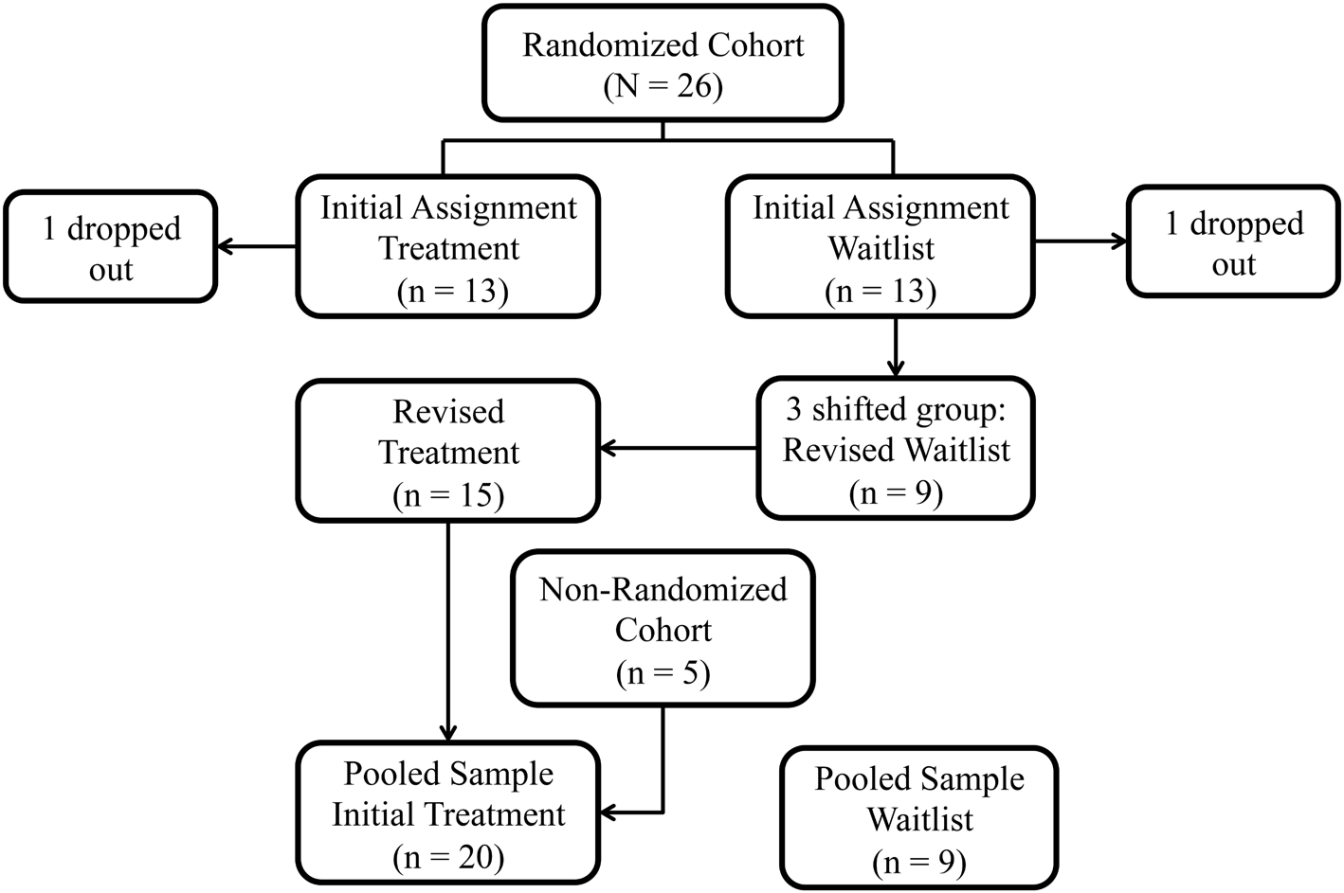
**Study group assignment: original randomization group assignments in the Randomized Cohort, subsequent shifts, and addition of the Non-Randomized Cohort**.

The study was conducted in two phases. Phase 1 was a randomized controlled trial (RCT). We enrolled 26 children in the Randomized Cohort on a rolling basis and assigned them to one of the two groups through the use of a random number sorter: Treatment (*n* = 13) or Waitlist-Control Group (*n* = 13) (Figure 1). Two children, one from each group, did not complete the treatment protocol and were dropped from further analyses. Three children originally assigned to the Waitlist-Control Group were reassigned to the Treatment Group to accommodate the children’s circumstances: one to complete the treatment protocol before the anticipated birth of a sibling, one to avoid delay in physician-prescribed botulinum toxin A injections, and one to avoid further waiting for a child whose participation was delayed while she enrolled in a physical therapy program. The revised sample size in the Randomized Cohort was Treatment Group *n* = 15 children and Waitlist-Control Group *n* = 9.

Phase 2 was an Open Label Extension. Children randomized to the Waitlist-Control Group received the treatment after the wait period. In addition, funding permitted the enrollment of five additional children (“Non-Randomized Cohort”), all of whom were assigned to the Treatment Group in order to complete the protocol before the end of the funding period. We then compared the Treatment Group (*n* = 20) to the Waitlist-Control Group (*n* = 9). We also evaluated the pooled sample (*n* = 27) for change over time.

**Figure 2:**
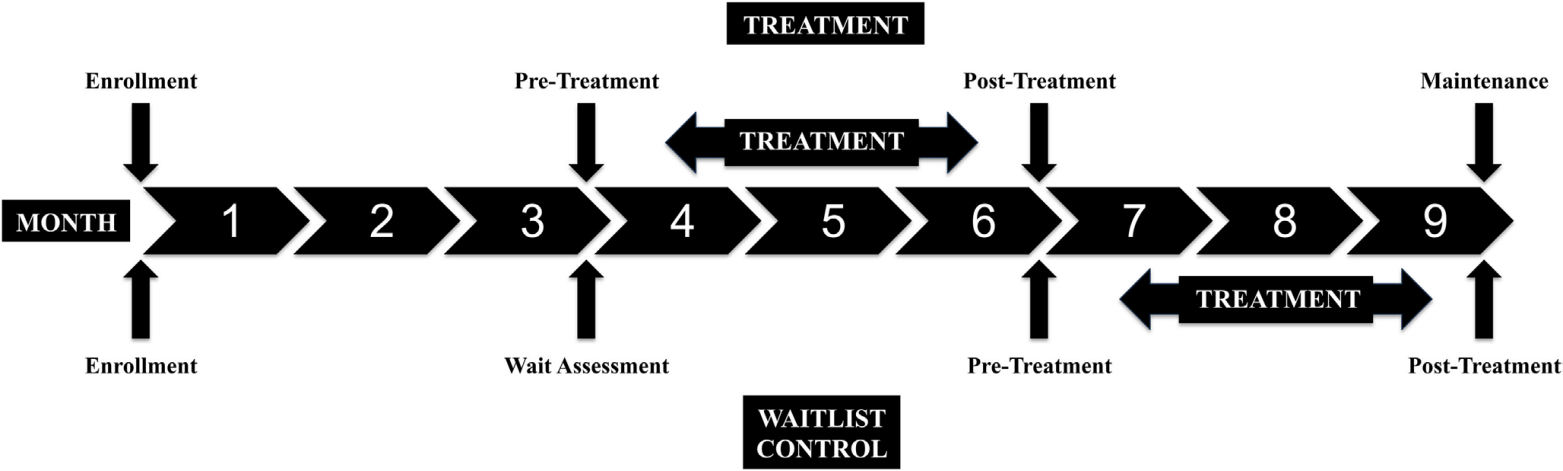
**Study design: assessment points and treatment periods for the Treatment and Waitlist-Control groups**.

All of the participants were scheduled for an evaluation at Enrollment and every 3 months for a total participation period of 9 months. Figure 2 shows the stages of the study. All of the children waited 3 months and had a second assessment prior to MSI treatment in order for us to assess the rate of developmental change prior to the experimental treatment. The second assessment was called the Pre-Treatment Assessment for the Treatment Group and the Wait Assessment for the Waitlist-Control Group (Figure 2). The Treatment Group underwent MSI therapy between the second and third assessments. The Waitlist-Control Group began MSI therapy after the third assessment. Both groups had a Post-Treatment Assessment and the Treatment Group was also assessed at a 3-month Maintenance assessment. All participants completed at least three assessments, including the post-treatment assessment, and 27 of the 29 children completed four assessments (one moved away from the geographic area before study completion and one began therapy with botulinum toxin A injections), to show whether skills remained stable during maintenance for the Treatment Group.

### Treatment

Each child was scheduled for 10 weekly 60- to 90-min sessions of the MSI intervention. All of the children completed 9 or 10 of the scheduled sessions. A single Certified Advanced Rolfer (KSP) with over 35 years of experience working with young children provided the therapy in her private studio. MSI treatments followed the specific, structured sequence that addressed the entire body. The researchers prepared the parents and the children by explaining the therapy as a type of bodywork similar to deep tissue massage. Children remained partially clothed and lay on a table, sat on the floor, or sat on a parent’s lap during the treatment. During each session, the clinician manually manipulated the muscles and fascia of the section of the body that was the focus for that day.

### Primary outcome measure: The gross motor function measure-66

An assessor (physical therapist or pediatrician) blind to each child’s group assignment completed the Gross Motor Function Measure-66 (GMFM-66). The GMFM-66 is an observational measure that was developed to capture the gross motor capacity of children with CP. It is has been shown to be valid, reliable, and responsive to change (19). The 66-item tool utilizes a 0–3 rating scale to measure skills in five different dimensions (A: Lying and Rolling, B: Sitting, C: Crawling and Kneeling, D: Standing, and E: Walking, Running, and Jumping). The total score, between 0 and 100 (higher scores indicating greater function), is obtained using specialized program scoring software that is based on a logit scale. Each assessment was videotaped and scored. After the conclusion of the study, videos for participants who demonstrated irregular score patterns were reviewed and their corresponding GMFMs rescored if required.

### Secondary outcome measure: GAITRite^®^ electronic walkway

Gait assessments of all ambulatory children were recorded and analyzed using the GAITRite^®^ electronic walkway. Its ease of operation makes this 14-foot walkway suitable for use with children. Several studies have documented the reliability of the measures in children with CP (20–22). As a child traverses the GAITRite^®^ walkway, pressure exerted by foot contact on the surface activates sensor pads embedded in the electronic mat. The walkway records the geometry of the activating foot contact and the relative arrangement of contact points in two-dimensional space. Spatial and temporal characteristics of the gait are calculated based on foot contact geometry.

Children walked barefoot over the walkway at a self-selected pace. Multiple passes over the walkway were obtained at each assessment. For analysis, a Walk was defined as the averaged data of the first two and up to four passes over the walkway that included a minimum of eight fluent steps in one direction across the mat with one foot always on the mat (indicating a walking pace). Passes in which the participants ran, stopped, or navigated off the mat were excluded.

### Secondary outcome measure: Parent satisfaction survey

At the conclusion of the study, parents were given a “Parent Satisfaction Survey” that the research team developed to record parents’ impressions of changes they saw in their children over the course of the study. The survey also allowed parents to provide feedback to the researchers about the study in general. The survey included the domains of emotional affect, functional body control, balance/flexibility, strength, height, weight, and functional achievements. The 11-question survey included open-response questions, such as, “Did you notice any changes in your child’s balance/flexibility (walking, standing, climbing stairs, tripping)?” and “Did you notice any changes in your child’s strength (self support, pulling up, climbing)?” The survey also included two ratings of satisfaction: “Parent Rating of Satisfaction” and “Parent Rating of Child Satisfaction.” Each rating was recorded on a Likert Scale, with 1 as the lowest score and 10 as the highest score.

### Data analysis

#### Primary Analysis

Analyses were conducted using SPSS version 22. As preliminary analyses, we first compared the Treatment and Waitlist-Control groups from the Randomized Cohort using independent-samples *t*-tests and Fisher’s Exact Test to establish if the groups were comparable on the basis of mean age, gender, race (comparing White to Non-White), CP type (comparing hemiparesis diagnosis to non-hemiparesis diagnosis), CP severity (comparing GMFCS levels I and II to III and IV), and mean GMFM-66 score at Enrollment. To assess treatment efficacy within the Randomized Cohort, we used a Repeated Measures ANOVA; GMFM scores at assessments II and III (Pre-Treatment to Post-Treatment for the Treatment Group and Wait Assessment to Pre-Treatment for the Waitlist-Control Group) were the within subject variables and randomization group was the between subject variable.

In the Open Label Extension, we compared the five children in the Non-Randomized Cohort to the group of 24 children in the Randomized Cohort using independent-samples *t*-tests and Fisher’s Exact Test to ensure that the groups remained comparable in composition on the same metrics as mentioned above. We then compared the expanded Treatment Group (*n* = 20) to the Waitlist-Control Group (*n* = 9) using Repeated Measures ANOVA, following the same strategy as in the RCT.

In order to examine whether there were time by group interactions at any point during the study, we completed the Repeated Measures ANOVA on the pooled sample of children who completed all four assessments (*n* = 27). For this analysis, GMFM scores at assessments I–IV were the within group variable and randomization group was the between group variable.

### Secondary analyses

For the secondary gait analysis, we selected a small set of dependent variables that captured different aspects of gait from the many inter-related measures that the GAITRite^®^ walkway generates, as follows: foot length and foot width, derived from the length and width of sensors activated by foot pressure progressing along the mat, and stride length, defined as the forward-progressing line between the heel points of two consecutive footprints of the same foot. Selected temporal parameters were as follows: double support time, defined as the percent of the gait cycle spent with both feet on the mat, and velocity, defined as distance traveled divided by ambulation time (23). Paired samples *t*-tests were performed to compare Pre-Treatment to Post-Treatment measurements. When differences were noted, paired *t*-tests were used to determine if differences were present between the assessment immediately prior to the Pre-Treatment Assessment (Enrollment for the Treatment Group and Wait Assessment for the Waitlist-Control Group) and Pre-Treatment and also between Post-Treatment and Maintenance Assessments.

For the Parent Satisfaction Survey, the mean scores for the two Likert scales of “Parent Rating of Satisfaction” and “Parent Rating of Child Satisfaction” were calculated from all available responses.

## Results

### Randomized controlled trial

Table 1 shows demographic and clinical characteristics of the children in the RCT. Within the Randomized Cohort, the Treatment Group (*n* = 15) did not differ from the Waitlist-Control Group (*n* = 9) in terms of mean age, gender, race, CP type, CP severity as measured by the GMFCS, and mean GMFM-66 score at Enrollment.

**Table 1:** **Demographic and clinical information on participants**.

	Randomized Cohort		Non-Randomized Cohort
	Waitlist-Control (*n* = 9)	Treatment^a^ (*n* = 15)	Treatment^b^ (*n* = 5)
Mean age, years (SD)	2.2 (0.8)	2.4 (1.0)	2.1 (0.7)
Male, % (*n*)	55.6 (5)	40.0 (6)	20.0 (1)
Non-White, % (*n*)	44.4 (4)	26.7 (4)	60.0 (3)
Type of spastic cerebral palsy, % (*n*)	Hemiparesis: 44.4 (4)	Hemiparesis: 33.3 (5)	Hemiparesis: 60.0 (3)
	Diplegia: 0.0 (0)	Diplegia: 20.0 (3)	Diplegia: 0.0 (0)
	Quadriparesis: 55.6 (5)	Quadriparesis: 46.7 (7)	Quadriparesis: 40.0 (2)
Gross Motor Function Classification System level, % (*n*)	Level 1: 11.1 (1)	Level 1: 20.0 (3)	Level 1: 40.0 (2)
	Manual ability	Manual ability	Manual ability
	Classification	Classification	Classification
	System	System	System
	Level 2: *n* = 0	Level 2: *n* = 1	Level 2: *n* = 0
	Level 3: *n* = 1	Level 3: *n* = 2	Level 3: *n* = 1
	Level 4: *n* = 0	Level 4: *n* = 0	Level 4: *n* = 1
	Level 2: 33.3 (3)	Level 2: 26.7 (4)	Level 2: 20.0 (1)
	Level 3: 0.0 (0)	Level 3: 20.0 (3)	Level 3: 0.0 (0)
	Level 4: 55.6 (5)	Level 4: 33.3 (5)	Level 4: 40.0 (2)
Mean Gross Motor Function Measure-66 score at Enrollment (SD)	37.9 (19.8)	40.6 (14.7)	40.3 (17.7)

*^a^Differences between Waitlist-Control and Treatment groups within the Randomized Cohort were not statistically different*.

*^b^Differences between the Randomized and Non-Randomized cohorts were not statistically different*.

The critical assessment of the efficacy of MSI treatment was change in GMFM scores between assessments II and III. There was a significant effect of time *F*(1, 22) = 8.096, *p* = 0.009, but no significant effect of group *F*(1, 22) = 0.394, *p* = 0.537 and no significant time by group interaction *F*(1, 22) = 0.911, *p* = 0.350.

### Open label extension

Table 1 documents that the five additional participants who constituted the Non-Randomized Cohort did not differ significantly from the group of randomized participants in terms of mean age, gender, race, CP type, severity of CP as measured by the GMFCS, and mean GMFM-66 score at Enrollment.

**Figure 3:**
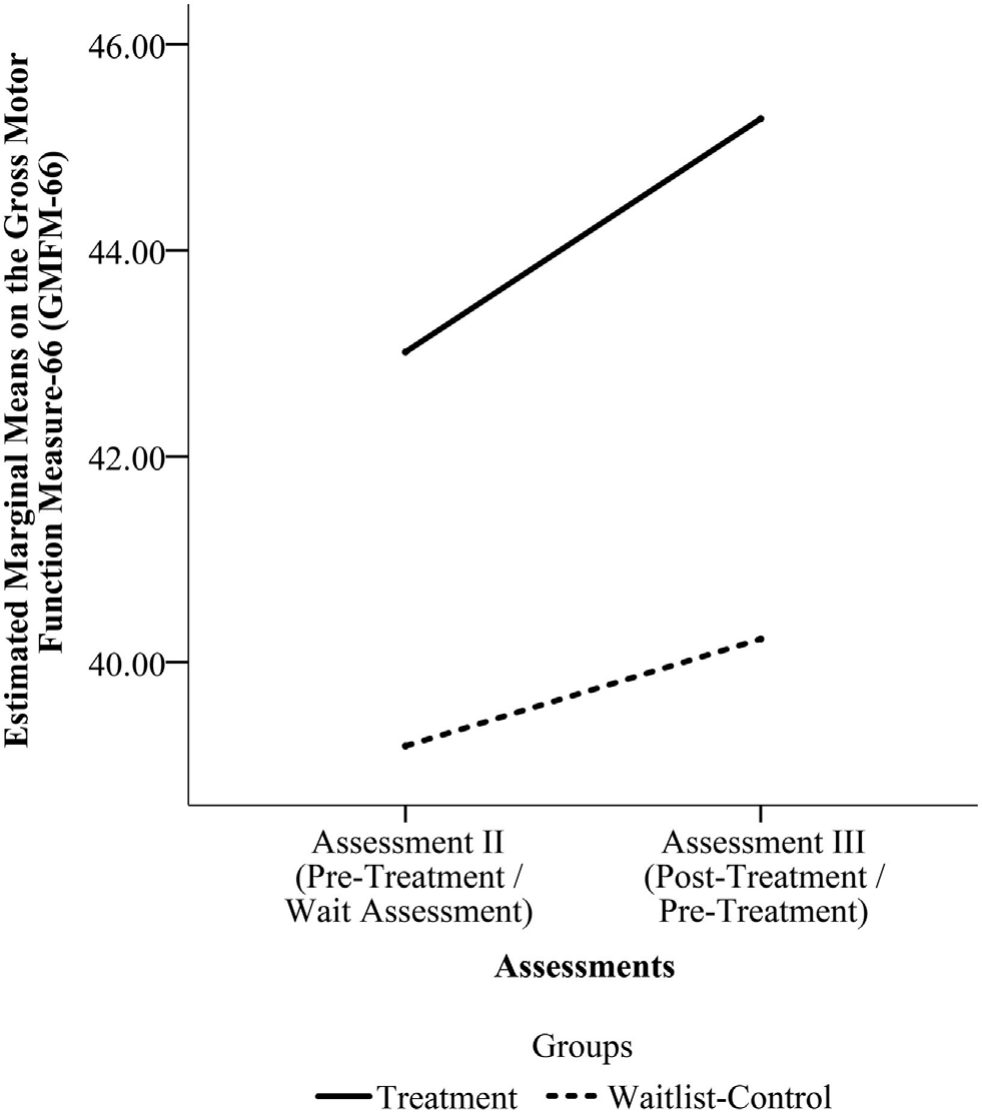
**Estimated marginal means on the Gross Motor Function Measure-66 (GMFM-66) from Assessment II to Assessment III for the pooled sample**. (Assessment II is the Pre-Treatment Assessment for the Treatment Group and the Wait Assessment for the Waitlist-Control Group. Assessment III is the Post-Treatment Assessment for the Treatment Group and the Pre-Treatment Assessment for the Waitlist-Control Group.)

With the larger sample (*N* = 29), the comparison of change in scores for the Treatment Group and for the Waitlist-Control Group from assessment II to III found a main effect of time *F*(1, 27) = 9.786, *p* = 0.004 but no effect of group *F*(1, 27) = 0.435, *p* = 0.515 or time by group interaction *F*(1, 27) = 1.349, *p* = 0.256. Figure 3 shows the estimated marginal mean scores for the GMFM assessments at the two time points.

**Figure 4:**
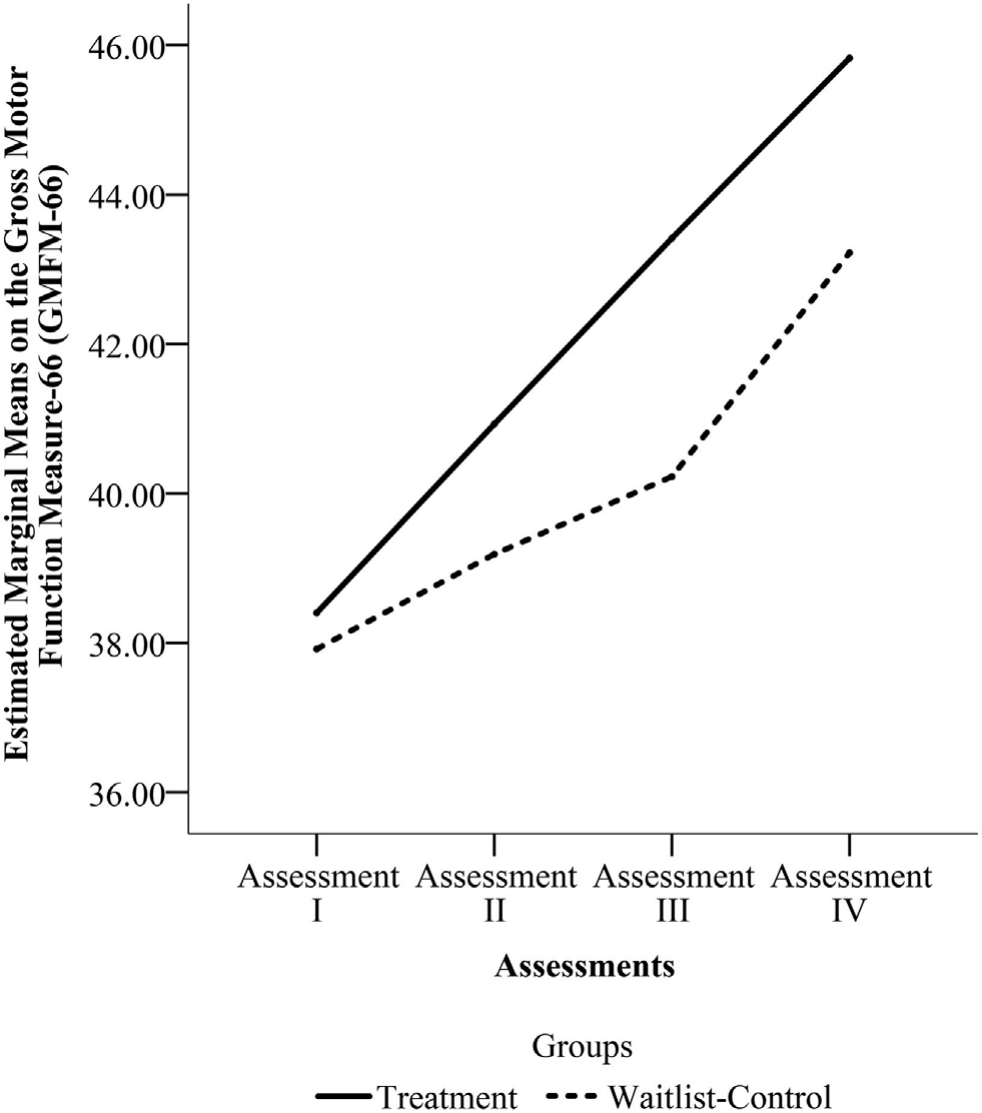
**Estimated marginal means on Gross Motor Function Measure-66 (GMFM-66) from Assessments I through IV for the pooled sample**. (Assessment I is the Enrollment Assessment for both groups. Assessment II is the Pre-Treatment Assessment for the Treatment Group and the Wait Assessment for the Waitlist-Control Group. Assessment III is the Post-Treatment Assessment for the Treatment Group and the Pre-Treatment Assessment for the Waitlist-Control Group. Assessment IV is the Maintenance Assessment for the Treatment Group and the Post-Treatment Assessment for the Waitlist-Control Group.)

The pooled sample that underwent four assessments (*n* = 27) was used to detect any differences in rate of change between the two groups from Enrollment to the final assessment. Mauchly’s Test indicated that the assumption of sphericity had been violated for the main effects of time, *x*^2^(5) = 23.458, *p* = 0.000. Thus, we used the Greenhouse–Geisser correction for degrees of freedom (*ϵ* = 0.615). There was a significant effect of time, *F*(1.845, 46.114) = 18.324, *p* = 0.000, but no significant effect of group, *F*(1, 25) = 0.090, *p* = 0.767 and no time by group interaction, *F*(1.845, 46.114) = 0.866, *p* = 0.420. Figure 4 shows the change across the four assessments for the two groups in the pooled sample.

### Secondary results: Gait parameters

Table 2 includes the mean values for the Pre-Treatment and Post-Treatment Assessments for each measure in comparison to normative data for children of comparable ages on those measures (22, 24). Two spatial parameters, foot width and stride length, and both temporal parameters were unchanged before and after treatment.

**Table 2:** **Mean gait parameters on more affected side in relation to normative data for ambulatory children with CP**.

Parameter	Normative data mean (SD)	Pre-Treatment mean (SD)	Post-Treatment mean (SD)	*t*	*p*
Foot length (cm)	16.0 (2.52)	12.1 (2.03)	12.6 (2.32)	−2.54	0.04
Foot width (cm)	7.0 (2.11)	5.8 (1.33)	6.1 (1.48)	−0.98	0.36
Stride length (cm)	68.8 (14.55)	62.8 (6.52)	64.1 (7.68)	−0.50	0.63
Double support time (% gait cycle)	16.5 (5.0)	21.9 (4.40)	20.1 (5.24)	0.95	0.38
Velocity (cm/s)	101.1 (29.9)	84.1 (11.40)	86.6 (14.51)	−0.47	0.65

**Figure 5:**
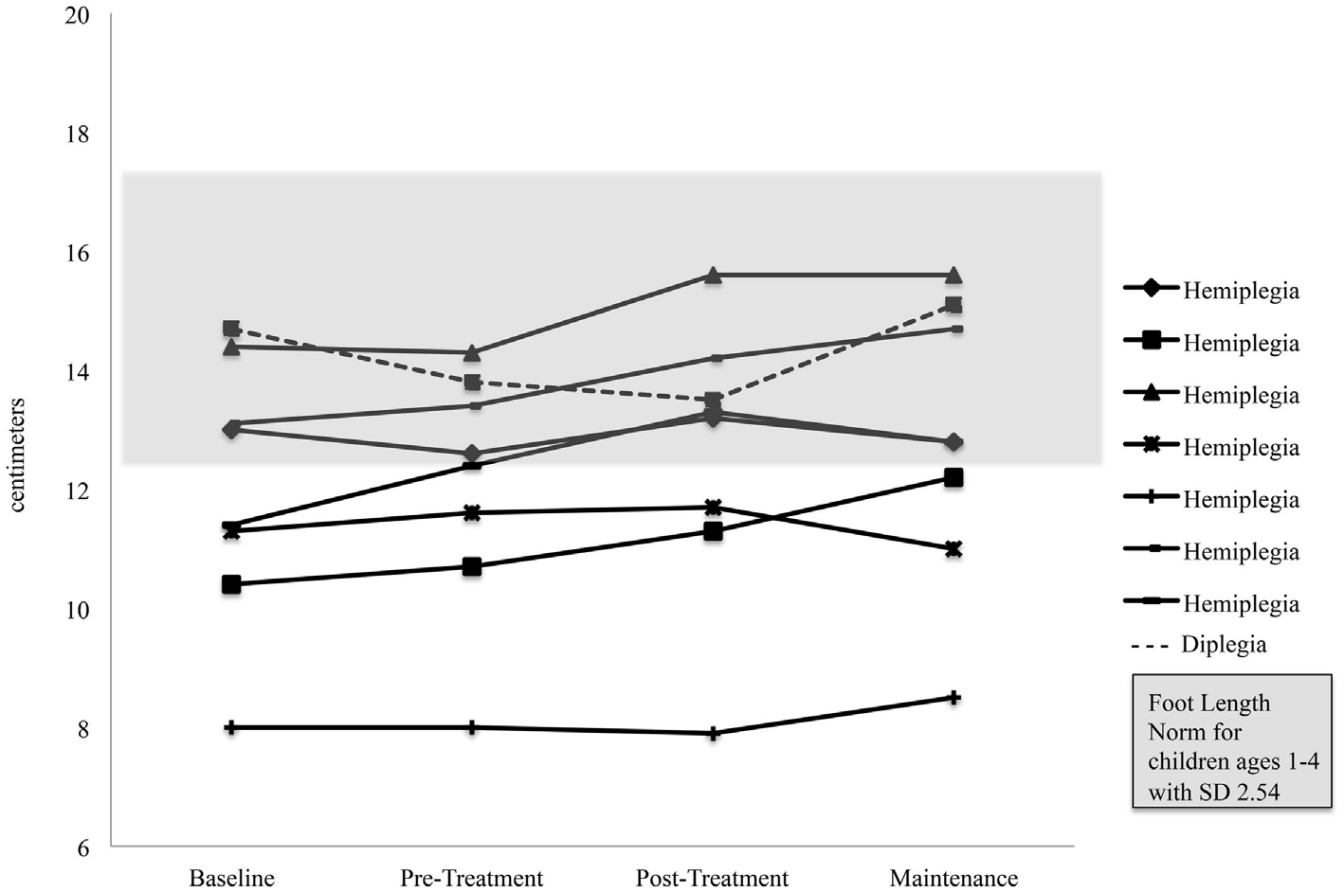
**Foot length on the more affected side of ambulatory participants with CP**.

Foot length on the more affected side increased significantly for the pooled sample between the Pre-Treatment and Post-Treatment intervals (*M* = 12.1, SD = 2.03) and (*M* = 12.59, SD = 2.32), *p* = 0.04. Figure 5 shows foot length of the more affected foot in study participants over time, with normative foot size for children in this age group referenced (24). Subsequent analyses found that foot length did not change between the assessment prior to Pre-Treatment (Enrollment for the Treatment Group and Wait Assessment for the Waitlist-Control Group) and the Pre-Treatment Assessment (*M* = 12.04, SD = 2.23) and (*M* = 12.1, SD = 2.03), *p* = 0.76 and were maintained at Maintenance (*M* = 12.8, SD = 2.35), *p* = 0.40. As shown in Figure 5, the effect was greatest in the children with hemiplegia; the change between Pre-Treatment and Post-Treatment for these seven children was significant (*M* = 11.86, SD = 2.06) and (*M* = 12.46, SD = 2.48), *p* = 0.02.

### Secondary results: Parent satisfaction survey

Parents of 25 of the 29 participants (86%) completed the Parent Survey. The mean “Parent Rating of Satisfaction” was high at 8.4/10 (minimum = 3.0, maximum = 10.0, SD = 1.9). The “Parent Rating of Child Satisfaction” was high at 8.6/10.0 (minimum = 3.0, maximum = 10.0, SD = 1.8).

Many parents reported observed qualitative improvements in their children’s gross motor skills. One parent wrote that her child experienced “improved walking, climbing stairs, sitting on floor cross-legged, decreased limp.” Another parent noticed that her child held his “left hand less fisted.” A common comment was that the children enjoyed the massage. One parent said, “It felt so good to him like massage.” Another exclaimed, “Rolfing – she loved it! And we saw many gains during therapy.” Changes were also noted in subjective well-being and quality of life, such as “improved mood.” One parent reported that her child had a “more relaxed demeanor[,] improved sleep[, and was] calmer for longer periods of time.” Another parent concluded her survey by writing, “We will do it again in the future.”

## Discussion

### Summary of results

The aim of this study was to investigate the effect of MSI therapy on the gross motor function and gait of young children with CP. The results of both the RCT and Open Label Extension showed that the children improved in gross motor function over time, but the rate of improvement on this measure was no greater immediately after treatment than with developmental change alone. Analyses of spatial and temporal gait parameters using an electronic walkway revealed changes in foot length, possibly representing improved heel strike, on the more affected side but no changes in the other gait parameters. A parent survey at the conclusion of the study found that parents were generally very satisfied with the treatment and saw improvements in their children’s function that were not detected by the selected outcomes measures. In summary, in this sample of children with CP, MSI did not increase the rate of motor skill development, but was associated with an improvement in gait quality and with positive parent reports of changes.

### Lack of change in GMFM-66 scores as a function of treatment

We did not detect changes in the rate of improvement on the GMFM as the result of MSI treatment. These negative findings suggest that MSI treatment is not effective in improving the rate of gross motor development in children with CP. However, the secondary analyses suggested modest qualitative improvements. It is, thus, possible that the treatment led to changes that were not detected by the GMFM-66 measure.

The GMFM is a very demanding outcome measure. Studies of other therapies have also failed to reach significant change on the broader 88-item GMFM measure (25). Studies assessing botulinum toxin A injections have found improvements in spasticity reduction and gait improvements without corresponding statistical difference in GMFM scores between treatment and control groups (26–29), did not include a control group, or did not compare the GMFM improvement to that expected with time alone (30, 31). Of over 100 studies, we found only three RCT studies that showed improvements in GMFM scores after treatment with botulinum toxin A compared to controls (32–35). For future studies, other outcomes measures of gross motor skill that may be more sensitive to change than the GMFM-66 should be considered.

Why might the GMFM fail to register change as a result of therapy in our study population? The assessment requires cooperation from the children during testing. Young children with CP may be inconsistent in their behavior and motivation and, therefore, inconsistent in their performance on the measure. In this study, most children showed consistent improvement in their GMFM-66 score. However, occasionally, children experienced a reduction in their scores from session to session. Many factors, such as mood, sleep disturbance, and level of cooperation, may contribute to the variability in scores (36). For children who are either at the floor or the ceiling of the skills that the GMFM is able to measure, a very slight change in consistency in the child’s performance may bring about a dip in performance.

Another challenge in using the GMFM-66 as the outcome measure is that the degree of expected change varies as a function of age and severity of motor disability. In previous trials, children who were young (<3 years of age) and had mild CP showed greater change in GMFM scores than children who were older and had more severe CP (19). Because the children in our study were young, the rate of developmental change, even without treatment, was relatively rapid, making it difficult to discern changes specifically related to the intervention. An acknowledged limitation of the GMFM measure is that children functioning in the middle of the GMFM-66 range have greater potential to change than children whose initial assessment is either very low or very high. In this study, 6 children were at GMFCS Level I and 12 children were GMFCS Level IV, with 18 out of 29 total participants in either of one of these categories. In both of these groups, it would be difficult to detect change based on the treatment. Because of the heterogeneity of the etiology and clinical presentation amongst children with a diagnosis of CP, Damiano recently cautioned that in order to advance evidence-based practice in the field of CP treatment, investigators and clinicians must consider each child individually when determining a treatment’s potential to improve function, and when developing and interpreting RCTs, answering not only the question of “what works” but also the question of “what works for whom” (37).

A final challenge in using the GMFM-66 as the outcome measure was that it was designed to measure change in skill level rather than change in quality of movements. As stated by Russell in a validation study for an early version of the GMFM, “It is likely that the changes detected by the GMFM reflect only part of the ‘real’ change in motor behavior over time, since many aspects of improvement are qualitative rather than quantitative. For example, we have the impression that when children use aids to perform motor functions they often have more qualitative than quantitative improvement in function. The GMFM does not appear to detect these changes or improvement well” (38). Indeed, in order to achieve a higher score on the GMFM-66, children must make relatively major changes in gross motor function. For example, a child with hemiplegia may have a markedly improved heel strike after treatment, contributing to stability and balance. To observe changes on the GMFM-66, however, she might have to make the significant gain of being able to stand on her affected leg and kick a ball with her other foot. Such an accomplishment might exceed the type of change that the improved heel strike might be expected to induce.

### Changes in the gait analyses after treatment

The quasi-experimental gait analysis demonstrated a significant increase in foot length on the more affected side after treatment. We interpreted increased foot length as an increase in the amount of foot contact with the mat. We did not think that the change represented growth in the foot because the less affected foot did not change significantly in length and both feet did not change in width, as would be predicted with growth. Rather, the selective increase in foot length most likely represents a lowering of the heel toward the floor and decreased toe walking, resulting in greater overall foot contact with the mat. We do not think that this finding was a spurious result. We did not find a change in foot length prior to treatment, in the maintenance phase, or in the foot on the less affected side. Improvements in heel strike could be seen visually on the videotapes obtained during the use of the GAITRite^®^ electronic walkway. Finally, some parents mentioned on the final survey that they had observed an improvement in gait and balance. It is also interesting to note that three children began to walk during or shortly after MSI. One child started walking within a few months of completing the treatment. These data suggest that MSI, when used as a complementary treatment, may improve aspects of gait in ambulatory children with mild spastic CP.

Ambulatory children with spastic CP demonstrate specific gait abnormalities. Ankle plantar flexor muscles are often overactive with ineffective dorsiflexor muscles, leading to the dynamic equinus foot deformity and poor heel strike, or incomplete foot contact, characteristic of toe walking. Decrease in dynamic equinus can be a treatment goal because it contributes to balance, stability, and reduces energy expenditures during walking, leading to functional improvements. The change in foot length occurred despite the fact that MSI treatment encompassed the whole body and did not specifically address only the foot. Targeting a specific part of the body may not be necessary for change to occur. It is interesting to note that this improvement in a motor skill would *not* be quantified in the GMFM-66, supporting the notion that MSI may improve quality of movement rather than promote quantitative functional change.

Two previous studies of MSI and gait found improvements in gait that we did not detect. A 10-child case series using bilateral insole sensors to assess gait parameters found that children with mild CP demonstrated improvements in cadence, velocity, and stride length; children with moderate CP demonstrated only improvements in velocity; and ambulatory children with severe CP did not demonstrate any gait improvements (15). Because of our small sample size, we did not analyze our ambulatory children separately based on CP severity, and, therefore, we may not have picked up on any differences within individual severity subgroups as the earlier study did. A recent case series using similar methodology as the current study in two older children with spastic CP found improvements in cadence and double support time after a course of MSI (17). Unlike the previous study, we analyzed only passes over the mat in which one foot was on the mat at all times in order to eliminate runs. Velocity independently influences cadence and time spent in single support and double support for children with CP (39). We chose to limit gait measurement variability by eliminating runs. However, we may have limited our ability to detect increases in maximum velocity, and its correlate, cadence. Likewise, because a running child is likely to spend less time in double support than a walking child, we may have been less likely to find changes for this gait parameter in our walking sample.

### Results of the parent survey

The qualitative reports from parents of the participants suggested that the children benefited from the therapy in ways not quantified by the Gross Motor Function Measure-66. Parent comments included “better walking, balance still shaky but better,” “better head control,” “improved functional use of hands,” and “decreased limp.” Such comments highlighted improvements in the quality of the children’s movements rather than in motor skills that would be measured by the GMFM-66. Such qualitative changes may contribute to the children’s ability to engage in day-to-day tasks. We recognize that parents were aware of which treatment phase the child was in over the course of participation in the study.

### Limitations

Limitations of the study were the relatively small sample size of 29 children and the uneven distribution of GMFCS levels. In addition, the Treatment and Control groups were weighted toward more severe levels of CP, which may have limited the amount of improvement we were able to observe with the GMFM-66. Furthermore, because of scheduling conflicts and the late addition of five participants into the Treatment Group without randomization, our Treatment Group and Waitlist-Control Group were unequal in size, although similar in other variables. In the secondary gait analysis, because of the small study sample size, there were not enough ambulatory participants to utilize the RCT design of the larger study for the analyses, so a quasi-experimental design was used. We did not have any measures of motor function other than the GMFM-66, which is not a sensitive measure for detecting improvements in quality of movement. Similarly, the GAITRite^®^ electronic walkway may not be the optimal modality for determining improved heel strike or other gait improvements; metrics reflecting ankle kinetics, kinematics and use of in-shoe foot sensor technology might yield greater precision and accuracy. We did not have any measures that assessed spasticity reduction in our children. Another limitation is that the young participants were inconsistent in their performance on the GMFM assessments and in their gait assessments. We reported inconsistent performances on the GMFM assessments for some children and eliminated many passes across the GAITRite^®^ mat because of erratic ambulation. This variability and these exclusions may have limited our ability to observe changes.

## Conclusion and Future Directions

In summary, MSI treatment used as a complementary treatment in young children with spastic CP did not increase the children’s rates of change on the GMFM-66 beyond the background rate of change from development and other therapeutic interventions. In ambulatory children, the treatment was associated with increased foot length on the affected side, possibly representing improvements in heel strike and gait quality. Parents generally found MSI therapy to be beneficial to their children in ways that were not captured by the main outcome measure. We think that further assessment of manipulative therapies to address the muscle and extracellular matrix changes in spastic CP is warranted. Future studies might consider inclusion of other outcomes measures in the research design, including ones that measure quality of movement, spasticity, quality of life, and functional changes in domains, such as mobility, self-care, communication, and social function.

## Ethical Approval

All procedures were approved by the Stanford Institutional Review Board (Protocol 16807), and the recommendations were adhered to. Parents provided written informed consent.

## Conflict of Interest Statement

The authors certify that no party having a direct interest in the results of the research supporting this article has or will confer a benefit on them or on any organization with which they are associated.

## Funding

This study was supported by generous funding from the Gerber Foundation, Grant number 11PH-010-1210-2986 and from a Leadership Education in Developmental-Behavioral Pediatrics from the Maternal and Child Health Bureau, Health Resources and Services Administration, Grant number 77MCO9796-01-00.
